# Patient Perceptions of Drive-through Medical Treatment Facilities During the COVID-19 Pandemic

**DOI:** 10.5811/westjem.2021.3.51319

**Published:** 2021-08-17

**Authors:** Sean Stuart, Sally Mandichak, Julianne Davison, Shai Ansell, Timothy Parker

**Affiliations:** * Naval Medical Center Portsmouth, Department of Emergency Medicine, Portsmouth, Virginia; † Naval Medical Center Portsmouth, Department of Internal Medicine, Portsmouth, Virginia; ‡ Uniformed Services University of the Health Sciences, Department of Military and Emergency Medicine, Bethesda, Maryland

## Abstract

**Introduction:**

The cumulative burden of coronavirus disease 2019 (COVID-19) on the United States’ healthcare system is substantial. To help mitigate this burden, novel solutions including telehealth and dedicated screening facilities have been used. However, there is limited data on the efficacy of such models and none assessing patient comfort levels with these changes in healthcare delivery. The aim of our study was to evaluate patients’ perceptions of a drive-through medical treatment system in the setting of the COVID-19 pandemic.

**Method:**

Patients presenting to a drive-through COVID-19 medical treatment facility were surveyed about their experience following their visit. An anonymous questionnaire consisting of five questions, using a five-point Likert scale was distributed via electronic tablet.

**Results:**

We obtained 827 responses over two months. Three quarters of respondents believed care received was similar to that in a traditional emergency department (ED). Overall positive impression of the drive-through was 86.6%, and 95% believed that it was more convenient.

**Conclusion:**

Overall, the drive-through medical system was perceived as more convenient than the ED and was viewed as a positive experience. While representing a dramatic change in the delivery model of medical care, if such systems can provide comparable levels of care, they may be a viable option for sustained and surge healthcare delivery.

## INTRODUCTION

The coronavirus 2019 (COVID-19) pandemic introduced multiple new stressors on an already struggling and overburdened healthcare system. At the forefront of the pandemic, emergency departments (ED) had to absorb this new load. The sheer burden of disease, 6.3 million cases in an 8.5-month time frame,^1^ highlighted potential challenges in providing and delivering quality patient care. These hurdles included large patient volumes, various clinical presentations of the disease, the financial burden of medical resources and supplies, and maintaining staff safety in the face of a droplet-based infectious disease.

The cumulative burden of the COVID-19 virus on the US healthcare system is substantial. Complicating the picture is the fact that the severe acute respiratory syndrome coronavirus 2 (SARS-COV-2) spreads via respiratory droplet transmission^2^ and many patients are asymptomatic vectors of the disease. Given these characteristics, major cities such as New York City, Chicago, and Los Angeles were experiencing record case numbers in their EDs and hospitals.^2^ The burden falls on EDs to identify and isolate patients at risk while maintaining efficiency and safety for all patients and staff.^3^ Novel solutions have included telehealth visits and screening test facilities that include outdoor and drive-through venues, aimed at minimizing contact exposure and diverting less ill patients from the ED. Remote and drive-through COVID-19 screening facilities have become common place mechanisms that allow for the rapid testing of populations. Initial data from Korea demonstrated that such systems for COVID-19 are a feasible and efficient option for screening, testing, and counseling stable patients.^4^ However, most of the facilities are primarily for point-of-care testing, without the ability to evaluate and treat ill patients. To our knowledge few systems have expanded these drive-through systems to allow full clinician evaluations. Such systems represent a significant deviation from traditional healthcare delivery models.

While data is being collected on the systemic advantages of a pandemic screening system, there is limited data of the efficacy of such models and none assessing patient comfort levels with this change in healthcare delivery. The aim of our study was to evaluate patients’ perceptions of a drive-through medical treatment facility (DMEF).

## METHODS

Naval Medical Center Portsmouth (NMCP) is a 298-bed federal, academic hospital with nine branch clinics and an ED census of 86,000 annually. In response to the COVID-19 pandemic, NMCP’s ED established a DMEF in proximity to the ED. All adult patients presenting to the ED with symptoms of potential COVID-19 etiology and deemed non-critical were directed to the DMEF for initial evaluation.

### Drive-through Medical Treatment Facility Logistics

The DMEF was designed to allow full evaluation, dispositioning and treatment of outpatient patients with potential COVID-19 symptoms. It was staffed Monday through Saturday, 9 am – 4 pm, by an emergency physician who oversaw up to four advanced practice providers (APP), each with a corpsman (medical assistant) and one nurse per APP pair. The facility consisted of three 40’ × 50’ temporary shelters erected in a parking lot adjacent to the ED. These structures allowed patients to drive their vehicles through, and the entire medical process was handled while the patients remained in their vehicles. On arrival patients were screened by a triage nurse using a pre-made screening form to determine appropriateness for DMEF evaluation vs diversion to the main ED. If appropriate, the patient was then registered, vital signs were recorded, and a paper medical chart was prepared. The patients then drove forward to a treatment station where a history and physical exam were conducted. Select point-of-care testing for COVID-19, influenza, and group B strep were also available. Upon completion of the evaluation and disposition, the standard discussion of diagnosis, treatment and follow-up plans occurred aided by preprinted discharge forms. Select medications including antipyretics and common “cold medications” formulations (guaifenesin, dextromethorphan, etc) were available for immediate dispensing, with traditional paper prescriptions used for other indicated medications.

**Population Health Research Capsule**

What do we already know about this issue?

*Non-traditional healthcare delivery systems have been utilized in the setting of coronavirus disease 2019 to extend healthcare resources and mitigate transmission with limited data on patient perceptions*.

What was the research question?

*What are patients’ impressions of medical care delivered via a drive-through treatment facility?*

What was the major finding of the study?

*Patients overall had positive impressions of medical care delivered via a drive-through system*.

How does this improve population health?

*These findings suggest nontraditional healthcare delivery mechanisms can be well received by patients, and their utility should be further explored to optimize medical system coverage*.

### Patient Perceptions

**Figure 1:**
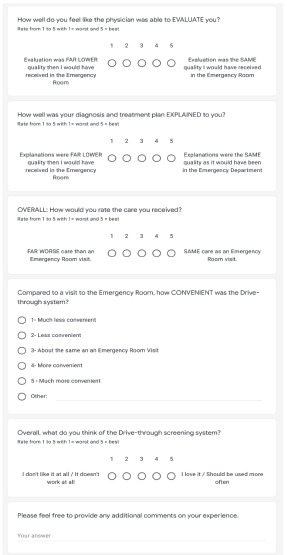
Patient perception survey of a drive-through medical evaluation system.

We developed a three-part questionnaire to evaluate patient perceptions of a drive-through medical system. The questionnaire was piloted with a small group of professionals (two physicians, two nurses, two administrative personnel) to ensure clarity of the survey questions. To optimize feasibility and participation, the final questionnaire was limited to five questions, each using a five-point Likert scale. (Figure 1). Three questions pertained to perceptions of components of their care (clinician evaluation, explanation, and level of care delivered), one assessed convenience, and one the overall impression of the use of drive-through systems for medical evaluation. An optional free-response section was included to allow participants to provide additional comments.

All patients completing medical evaluation at the NMCP’s DMEF were eligible to participate in the study. We excluded from participation any patients sent to the ED for further evaluation by DMEF providers. A convenience sample of patients from May 1–July 1, 2020 between 8 am – 4 pm were offered the opportunity to participate anonymously in the survey to evaluate their experience following their medical evaluation. Participants completed the survey via a provided electronic tablet. We examined all the data obtained and coded the responses. The data was then descriptively analyzed, and an appropriate test was applied in Excel (Microsoft Corp., Redmond, WA).

## RESULTS

**Figure 2:**
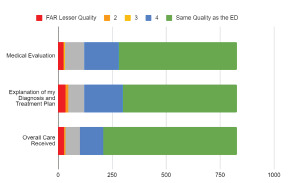
Patients’ perception of the quality of care received compared to their expected care in the emergency department.

Between May 1–July 1, 2020, we received a total of 827 responses. Given the anonymity of the survey, comprehensive data on demographics and comorbidities of respondents is not available. For the 2437 all comers to the DMEF, the median age was 32.5 (range 18–56 years old), and represented both active duty military and their dependents. Of the participants, 68% were male. For patient perceptions of the components of their care, three-quarters of respondents (n = 617) believed the overall care they received was equivalent to what they would have received in the ED with an additional 13.1% (n = 108) rating their overall care as similar (Figure 2).

**Figure 3:**
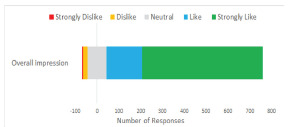
Overall patient impression of drive-through systems for medical evaluations.

**Figure 4:**
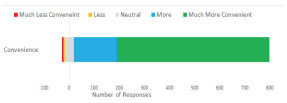
Patients’ impression of the convenience of the drive-through medical system compared to an emergency department visit.

A total of 86.6% (n = 715) of respondents gave positive overall impressions of the drive-through screening system compared to 3.0% (n = 25) responding negatively (Figure 3). In regard to convenience, 95.2% (n = 779) viewed the drive-through system as “more convenient than going to the emergency department,” while 1.2% (n = 10) and 4.6% (n = 38) viewed it as “less” and “equivalently” convenient, respectively (Figure 4).

## DISCUSSION

The COVID-19 pandemic has placed further strain on a medical system already struggling with access-to-care issues. In addition to the potential burden of new disease, the challenge of how to deliver healthcare in a way that is both efficient and effective while minimizing transmission risk to both healthcare workers and patients poses a challenge. This challenge has contributed to the rapid growth of pre-pandemic healthcare delivery mechanisms such as telemedicine. A report by the US Department of Health and Human Services found that telehealth adoption increased by nearly 50% in primary care from January through early June 2020.^5^ Likewise, countless drive-through COVID-19 screening centers were erected to facilitate mass population testing. Only a limited number appear to have offered traditional medical evaluation. While there is increasing evidence demonstrating the feasibility of these systems, there is limited evidence evaluating the quality of care provided and no consensus as to how patients perceive these dramatic changes in their healthcare delivery.

Our study sought to aid in the understanding of how patients perceive medical care delivered in a drive-through venue. In our study, the vast majority of patients evaluated in our DMEF reported positive experiences as denoted by high marks in the areas of quality of provider evaluation, explanation of diagnosis and treatment plan, and overall level of care. Additionally, the DMEF was felt to be significantly more convenient than a visit to the ED. Overall, in our study the patients had positive impressions of the use of a drive-through system for medical evaluations.

Satisfaction studies have repeatedly found wait times to be a key component in a patient’s impression of their medical experience.^6^ Perhaps more noteworthy is evidence suggesting that increased wait time induced emotional disutility in already ill patients.^7^ This fourfold reduction in time was likely a prime contributor to the high ratings especially in the area of convenience. Interestingly, not only was convenience the highest rated item on the survey (mean 4.39/5), but even the vast majority of people who were not satisfied with other aspects of their care still positively endorsed the convenience of the drive-through system.

Patient satisfaction is a complex and multifactorial process. However, it alone does not validate the quality of medical care provided nor is it directly linked to outcomes.^8^ However, patient satisfaction has become an increasingly used proxy indicator of the quality of healthcare delivery. Since the late 1990s the Centers for Medicare and Medicaid (CMS) has mandated the use of Consumer Assessment of Healthcare Providers and Systems (HCAHPS) surveys. The CMS then ties reimbursement to performance on this survey. However, it has been mentioned before that there is a noticeable absence of a single question pertaining to whether a patient felt they received adequate medical care.^9^ Our questionnaire attempted a cursory look at this gap by addressing the patient’s perception of their medical evaluation, their treatment plan explanation, and overall level of medical care. Here we found that despite the non-traditional setting and method, patients still felt they were receiving comparable levels of care from the providers.

While all measures in our survey received positive responses, the lowest mean satisfaction value (4.33) was associated with the perception of providers’ explanations, which would entail diagnosis, expected course, return precautions, and follow-up planning. This correlates with the subjective comments as well: although predominantly positive, negative comments were largely centered on the patients not fully understanding what they should do next or their follow-up plan. While the unconventional setting of drive-through care may very well contribute to communication lapses, effective communication and transitions of care have been longstanding challenges in healthcare. In the 2020 CMS report on HCAHPS, transitions of care received by far the lowest overall ranking. Additionally, numerous studies have cited communication disconnects as a source of poor outcomes and periods of care transition as vulnerable periods.^8^ A consumer survey by Kyruus (Kyruus Inc., Boston, MA) found issues with communication during virtual appointments, in which less than half of respondents said they left their visits knowing what the next steps were.^11^

## LIMITATIONS

Our study was limited by survey anonymity preventing exact demographic assessment of our population and a smaller (five-question) questionnaire, which restricted the granularity of the data. These decisions were made pragmatically, as the surveys were conducted in a drive-through venue, to minimize the Hawthrone effect and in an effort to increase recruitment, as shorter questionnaires have been shown to result in higher response rates.^12^ We were able to pull demographics for all patients who drove through the unit and maintain that the large number and consistency of the responses still allows for overall assessment. The DMEF was designed exclusively for the evaluation and treatment of COVID-19/influenza-like illness and would not be appropriate for all medical conditions. Even with these limitations, we believe the findings provide useful initial insight into patients’ perceptions of vehicle-based healthcare models.

## CONCLUSION

While a drive-through medical facility represents a dramatic change in the delivery model of medical care, our study suggests these drive-through medical systems can be well received by patients. If such systems can provide comparable levels of care, they may represent a viable and critical option for sustained and surge healthcare delivery.

## References

[1] Centers for Disease Control and Prevention (2020). CDC COVID Data Tracker.

[2] Centers for Disease Control and Prevention (2020). COVID-19 overview and infection prevention and control priorities in non-US healthcare settings.

[3] The New York Times (2020). Covid in the US: latest map and case count.

[4] Chavez S, Long B, Koyfman A (2021). Coronavirus disease (COVID-19): a primer for emergency physicians. Am J Emerg Med.

[5] Kwon KT, Ko JH, Shin H (2020). Drive-through screening center for COVID-19: a safe and efficient screening system against massive community outbreak. J Korean Med Sci.

[6] US Department of Health & Human Services (2020). Medicare beneficiary use of telehealth visits: early data from the start of the COVID-19 Pandemic.

[7] Woolen S, Kazerooni EA, Wall A (2018). Waiting for radiology test results: patient expectations and emotional disutility. J Am Coll Radiol.

[8] Centers for Medicare and Medicaid Services Joint Commission Center for Transforming Healthcare, Improving Transitions of Care: Hand-Off Communications.

[9] Boissy Adrienne (2020). Getting to patient-centered care in a post–Covid-19 digital world: a proposal for novel surveys, methodology, and patient experience maturity assessment.

[10] Prakash B (2010). Patient satisfaction. J Cutan Aesthet Surg.

[11] Hutton D (2020). Amid COVID-19 pandemic telehealth patient satisfaction high could drive future access.

[12] Guo Y, Kopec JA, Cibere J (2016). Population survey features and response rates: a randomized experiment. Am J Public Health.

